# Spotted Fever: An Undercover Cause of Hemophagocytic Lymphohistiocytosis in the Immediate Postpartum

**DOI:** 10.1155/2022/3348393

**Published:** 2022-03-01

**Authors:** Karen Aida Ibarra Stone, Jose Gabriel Solis, Enrique Blanco-Lemus, Jose Malagón-Rangel, Guadalupe Gordillo-Perez

**Affiliations:** ^1^Hospital de Especialidades, Centro Medico Nacional Siglo XXI, Mexico city, Mexico; ^2^Hospital de Cardiologia, Centro Medico Nacional Siglo XXI, Mexico city, Mexico; ^3^Unidad de Investigación de Enfermedades Infecciosas y Parasitarias, Centro Medico Nacional Siglo XXI, Mexico City, Mexico

## Abstract

Hemophagocytic lymphohistiocytosis (HLH) is characterized by a dysregulated activation of the immune system that causes fever, cytopenias, organomegalies, and hemophagocytosis. There are infectious, neoplastic, rheumatologic, and miscellaneous causes. Rickettsioses are a neglected cause of HLH. We report a confirmed case of an immunocompetent woman in Mexico with postpartum HLH secondary to spotted fever. We did a review of the literature for search of similar cases. The association between these two diseases was found in postmortem studies, unrelated to postpartum. This diagnosis should be considered in all patients with HLH without an evident cause in areas of epidemiological risk.

## 1. Introduction

Hemophagocytic lymphohistiocytosis (HLH) is characterized by a dysregulated activation of cytotoxic T lymphocytes, natural killer cells, and macrophages with a persistent inflammatory response. Mortality goes as high as 80%; HLH is classified as primary or secondary; this last can be subclassified into infectious, neoplastic, autoimmune, or miscellaneous [1].

Rickettsioses are a neglected cause of HLH. The genus *Rickettsia* contains more than 20 species, and the most representative are the spotted fever group, in which subspecies *R. parkeri, R. philipii, R. akari*, and *R. rickettsii* are closely related genetically [2, 3].

In Mexico, *R. rickettsii* has been identified in ixodid ticks causing large outbreaks of the disease with mortality rates from 30 to 80% [4, 5].

A vast majority of patients with Rocky Mountain spotted fever (RMSF) present the clinical triad of fever, headache, and rash. On the first 5 days of the illness, a fulminant disease can develop. Other spotted fevers are usually less severe although difficult to distinguish one from another [3, 4].

During pregnancy, RMSF and Mediterranean spotted fever (MSF) have been previously described with a higher risk of spontaneous abortions [6].

A healthy 32-year-old woman from Queretaro, Mexico, developed fever, headache, jaundice, and choluria 24 hours after the resolution of an uncomplicated pregnancy. Her past medical history was unremarkable. She referred having regular contact with dogs and farm animals. Pregnancy-related complications were excluded. Broad-spectrum antibiotics were administered, but her symptoms persisted; therefore, doxycycline was added due to clinical suspicion of zoonosis soon after she was referred to our internal medicine department.

**Figure 1:**
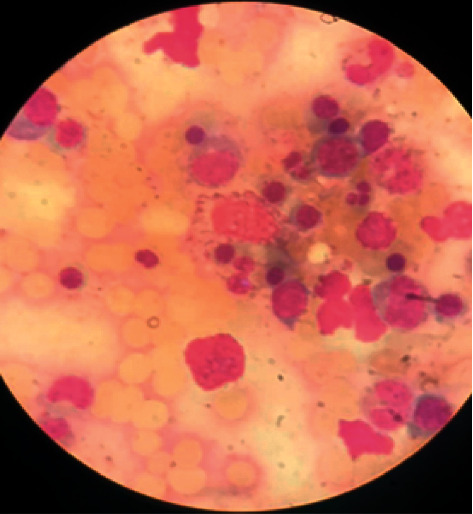
Bone marrow aspirate smear that shows hemophagocytosis.

**Figure 2:**
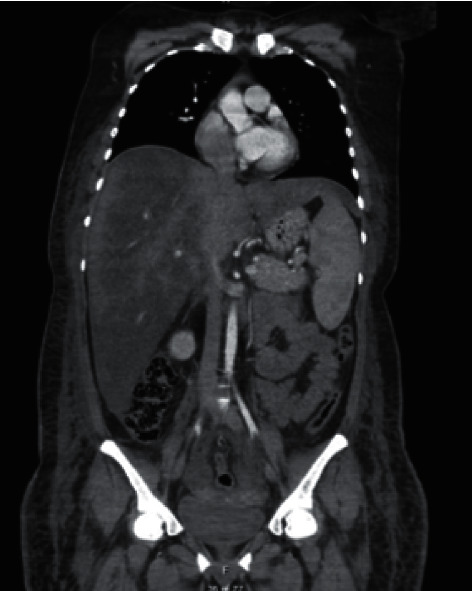
Computed tomography that shows massive hepatomegaly (29 cm) and splenomegaly (16 cm).

At her admission, she was ill-appearing and tachycardic. Physical examination showed a massive enlarged liver. Laboratory tests revealed pancytopenia (hemoglobin 8.2 g/dl, leucocytes 2840/mm^3^, and platelets 113000/mm^3^), hypertriglyceridemia (602 mg/dl), hyperbilirubinemia (6 mg/dl), elevated transaminases (aspartate aminotransferase 350 UI/L and alanine aminotransferase 58 UI/L) and lactate dehydrogenase (2419 UI/L), cholestasis (alkaline phosphatase 816 UI/L and gamma-glutamyl transferase 514 UI/L), coagulopathy, and extreme hyperferritinemia (49250 ng/ml). A bone marrow aspirate showed hemophagocytosis (Figure 1). Thoracoabdominal computed tomography (CT) reported massive hepatomegaly and splenomegaly (Figure 2). Based on HLH-04 criteria, the diagnosis was made.

Treatment based on the HLH-04 protocol was initiated using dexamethasone, ciclosporin, and intravenous immunoglobulin. Empiric therapy with meropenem, vancomycin, and doxycycline was instituted. The blood, urine, and bone marrow cultures were negative. Serologic studies for hepatitis B and C virus, human immunodeficiency virus, Epstein–Barr virus (EBV), toxoplasma, rubella, cytomegalovirus (CMV), *Brucella* spp., and *Borrelia burgdorferi* were negative. A PCR in blood for the detection of *Anaplasma phagocytophilum, Ehrlichia chaffeensis,* and *Babesia* and viral load for EBV and CMV were all negative. Negative antinuclear antibodies discarded rheumatologic diseases.

**Figure 3:**
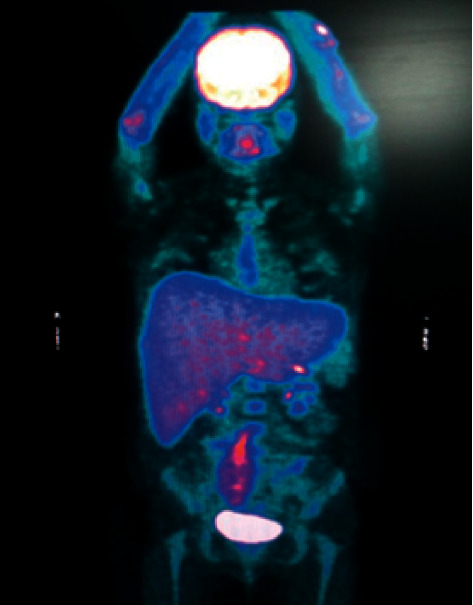
Positron emission tomography with 18-fluorodeoxyglucose that shows increased uptake in the liver.

For the diagnostic approach of malignancies, flow cytometric immunophenotyping of the bone marrow was normal. A positron emission tomography with 18-fluorodeoxyglucose showed increased uptake in the liver (Figure 3); consequently, an open biopsy was performed. The histopathological study revealed steatohepatitis with moderate necroinflammatory activity, focal fibrosis, and sinusoidal congestion, without evidence of lymphoma by immunohistochemistry. A nested PCR for *M. tuberculosis* on liver tissue was also negative.

A nested PCR assay to amplify the *rOmpA* and citrate synthase (*gltaA*) genes of spotted fever group rickettsioses was performed in blood and liver tissue by the use of primers previously described [5, 7] at a national reference center for tick-borne diseases with a positive result.

Treatment continued, and etoposide was added. During hospitalization, the patient presented considerable clinical and paraclinical improvement. She was discharged from our unit and has remained free of the disease after a 1-year follow-up.

Until now, few cases of HLH associated with rickettsioses have been reported, most of them associated with scrub typhus, MSF, or Japanese spotted fever [8, 9].

This is the first report of a patient with HLH secondary to spotted fever in the immediate postpartum. The only reported association between these diseases was found in 2 postmortem studies, where histopathological analysis of lymph nodes of patients with RMSF found hemophagocytosis in 8 children and 5 adults. Patients with other spotted fevers survive in most cases with early treatment [10].

In our case, the diagnosis was made with a nested PCR. This test can be used for the detection of the spotted fever group that shares the Rr190.547F and Rr190.701R conserved rOmpA primer sequences [7]. However, it was not possible to identify subspecies.

Remarkably, histopathology of the liver did not show abnormalities fully compatible with HLH or RMSF. This is probably due to early initiation of specific therapy [9].

In the same way, the absence of rash, an entity known as Rocky Mountain spotless fever, could be a result of early treatment [3].

In early infants, a rare HLH related to Chediak–Higashi syndrome (CHS), an autosomal recessive disorder characterized to join granules at the cell cytoplasm, with a distinctive clinical picture defined as oculocutaneous albinism and recurrent pyogenic infections, has been described. Akbayram et al. reported a 14-month-old girl who presented with HLH and pneumonia, observing giant intracytoplasmic granules in the bone marrow, the hair was noticeably finer and faded, and laboratorial analysis showed perforin-1, syntaxin-11, and UNC13D gene mutations diagnosing CHS [11]. This demonstrates the difficulty of finding the origin of HLH.

This report suggests that HLH could be a physiopathological mechanism in severe rickettsioses and that a high index of suspicion is required for the diagnosis. Treatment with doxycycline should be considered in all cases of HLH, especially when there is a compatible epidemiological background.
